# Automated patient-ventilator interaction analysis during neurally adjusted non-invasive ventilation and pressure support ventilation in chronic obstructive pulmonary disease

**DOI:** 10.1186/s13054-014-0550-9

**Published:** 2014-10-13

**Authors:** Jonne Doorduin, Christer A Sinderby, Jennifer Beck, Johannes G van der Hoeven, Leo MA Heunks

**Affiliations:** Department of Critical Care Medicine, Radboud University Medical Center, Geert Grooteplein Zuid 10, 6525 GA Nijmegen, The Netherlands; Department of Critical Care Medicine, St. Michael’s Hospital, 30 Bond Street, Toronto, ON M5B 1W8 Canada; Department of Pediatrics, University of Toronto, 555 University Avenue, Toronto, ON M5G 1X8 Canada; Keenan Research Centre for Biomedical Science of St. Michael’s Hospital, 30 Bond Street, Toronto, ON M5B 1W8 Canada

## Abstract

**Introduction:**

Delivering synchronous assist during non-invasive ventilation (NIV) is challenging with flow- or pressure-controlled ventilators, especially in patients with chronic obstructive pulmonary disease (COPD). Neurally adjusted ventilatory assist (NAVA) uses diaphragm electrical activity (EAdi) to control the ventilator. We evaluated patient-ventilator interaction in patients with COPD during NIV with pressure support ventilation (PSV) and NAVA using a recently introduced automated analysis.

**Methods:**

Twelve COPD patients underwent three 30-minute trials: 1) PSV with dedicated NIV ventilator (NIV-PSV_Vision_), 2) PSV with intensive care unit (ICU) ventilator (NIV-PSV_Servo-I_), and 3) with NIV-NAVA. EAdi, flow, and airway pressure were recorded. Patient-ventilator interaction was evaluated by comparing airway pressure and EAdi waveforms with automated computer algorithms. The NeuroSync index was calculated as the percentage of timing errors between airway pressure and EAdi.

**Results:**

The NeuroSync index was higher (larger error) for NIV-PSV_Vision_ (24 (IQR 15 to 30) %) and NIV-PSV_Servo-I_ (21 (IQR 15 to 26) %) compared to NIV-NAVA (5 (IQR 4 to 7) %; *P* <0.001). Wasted efforts, trigger delays and cycling-off errors were less with NAVA (*P* <0.05 for all). The NeuroSync index and the number of wasted efforts were strongly correlated (r^2^ = 0.84), with a drastic increase in wasted efforts after timing errors reach 20%.

**Conclusions:**

In COPD patients, non-invasive NAVA improves patient-ventilator interaction compared to PSV, delivered either by a dedicated or ICU ventilator. The automated analysis of patient-ventilator interaction allowed for an objective detection of patient-ventilator interaction during NIV. In addition, we found that progressive mismatch between neural effort and pneumatic timing is associated with wasted efforts.

## Introduction

Non-invasive ventilation (NIV) plays an important role in managing patients with acute respiratory failure, in particular in patients with chronic obstructive pulmonary disease (COPD). In patients with acute hypercapnic exacerbation of COPD, NIV improves outcome [1-3]. Accordingly, NIV utilization has increased over time among patients hospitalized for acute exacerbation of COPD, whereas the need for intubation has declined [2]. Despite these positive reports, some patients treated with NIV fail and require invasive mechanical ventilation [3,4]. Poor patient-ventilator interaction may contribute to NIV failure [5,6]. Delivering synchronous non-invasive assist is challenging with flow- or pressure-controlled systems [7], especially when using excessively leaky or highly compliant interfaces [8]. Using ventilators not dedicated to NIV, up to 46% of patients exhibit severe asynchrony [9]. The introduction of dedicated NIV ventilators and NIV algorithms in ICU ventilators improved patient-ventilator interaction, yet their performance varies and the inherent limitations of using flow or pressure to control assist remain [10].

As recommended [11,12], patient-ventilator interaction can be evaluated by using the diaphragm electrical activity (EAdi) [13]. Besides its monitoring capabilities, EAdi is used during neurally adjusted ventilatory assist (NAVA) as a controller signal for ventilatory assist [14]. Recent studies in heterogeneous groups of critically ill patients show that non-invasive NAVA (NIV-NAVA) improves patient-ventilator interaction relative to non-invasive pressure support ventilation (NIV-PSV) [15-18].

To our knowledge, there are no studies of patient-ventilator interaction strictly in COPD patients receiving NIV-NAVA, while these patients are more likely to exhibit severe patient ventilator asynchrony [19]. In addition, no study has used the EAdi signal to evaluate patient-ventilator interaction with dedicated NIV ventilators. Moreover, a new automated analysis method has recently been introduced in this journal for quantifying patient-ventilator interaction in a standardized fashion [20]. This automated analysis allows detection of asynchronies, such as wasted efforts, but also makes it easy to detect the more subtle dyssynchronies, such as trigger delays and cycling-off errors. The present study is the first to use this analysis method to quantify patient-ventilator interaction during non-invasive ventilation.

For the above-stated reasons, the aim of the present study was to evaluate patient-ventilator interaction, using an automated analysis, in COPD patients with NIV-PSV delivered by a dedicated NIV ventilator, and NIV-PSV and NIV-NAVA delivered by an ICU ventilator.

## Materials and methods

### Study subjects

Adult patients with acute respiratory failure and a medical history of COPD, admitted to the ICU for non-invasive ventilation were eligible for inclusion in the study. Patients with a known neuromuscular disorder, severe hypoxemic failure (PaO_2_/FiO_2_ < 100 mmHg), or hemodynamic instability requiring high-dose norepinephrine (>0.5 μg/kg/min) were excluded. The study was approved by the ethics committee of the Radboud University Medical Center (NL33351.091.11) and is in accordance with the ethical standards laid down in the 1964 Declaration of Helsinki and its later amendments. All patients gave their informed consent prior to the study.

### Study design

All patients undergoing NIV in our hospital receive a nasogastric tube to allow adequate feeding and prevent gastric hyperinflation. COPD patients undergoing NIV receive a nasogastric tube with a multiple array of electrodes placed at the distal end (NAVA catheter, 12 French; Maquet Critical Care, Solna, Sweden). Correct positioning was established by use of dedicated software. After enrollment and clinical stabilization, each patient received three 30-minute ventilation protocols in the following order:

1. PSV with the BiPAP Vision (Philips Respironics, Best, The Netherlands), a dedicated NIV ventilator, with pressure support and positive-end expiratory pressure (PEEP) levels set by the treating physician (NIV-PSV_Vision_).
2. PSV with the Servo-I (Maquet Critical Care, Solna, Sweden, NIV software v3.0), an ICU ventilator with NIV algorithm, with similar PEEP and pressure support levels (NIV-PSV_Servo-I_).
3. NAVA with the Servo-I (Maquet Critical Care, Solna, Sweden, NIV software v3.0), where NAVA level was adjusted to match peak pressure of NIV-PSV, using manufacturer-supplied software (NIV-NAVA).

BiPAP Vision uses the Auto-Trak Sensitivity algorithm to trigger and cycle off the ventilator and cannot be set individually. With NAVA, the back-up mode for triggering was set at flow triggering. In order to reduce the amount of leakage on ventilator performance, we chose to use a tightly fitted oronasal mask (Respironics PerforMax, Philips, Best, The Netherlands), a frequently used interface [21]. Switching between ventilators required modifications in measurement setup and short disconnection of the patient from the ventilatory circuit. In order to minimize discontinuation of assist, the order of interventions were not randomized.

At the end of each ventilator mode, respiratory discomfort was scored by use of a Visual Analog Scale (from 0 mm (no discomfort) to 100 mm (maximal imaginable discomfort)) and arterial blood gas analysis was performed from an indwelling arterial line.

### Data acquisition

Flow, airway pressure (Paw) and EAdi were acquired from the serial port of the Servo-I at a sampling rate of 62.5 Hz and recorded using dedicated acquisition software (Neurovent Research Inc., Toronto, ON, Canada). The BiPAP Vision does not have a data output port. Therefore, flow was acquired by placing a pneumotachograph (Fleisch no. 3, Phipps & Bird, Richmond, VA, USA) between the airflow port of the ventilator and its tubing, and Paw was acquired by placing a connection piece between the end of the tubing (after the leakage port) and the face mask, connected to a pressure transducer (range ±50 kPa, Freescale Semiconductor, Tempe, AZ, USA). Both Paw and flow were recorded at a sampling rate of 62.5 Hz and synchronized with the EAdi using dedicated acquisition software (Neurovent Research Inc., Toronto, ON, Canada).

### Data analysis

Study parameters were calculated from a stable 5-minute period at the end of each mode on a breath-by-breath basis using a software routine developed for Matlab (Mathworks, Natick, MA, USA). Measuring tidal volume by expiratory flow integration is not precise in the presence of leaks, therefore, tidal volumes are not presented in the manuscript. Neural respiratory rate was calculated as the number of EAdi peaks/min.

Patient-ventilator interaction was evaluated by comparing Paw and EAdi waveforms with automated computer algorithms [20]. Trigger and cycle-off error (that is dyssynchrony) were calculated as percentages of neural inspiratory and expiratory time periods, respectively. Events where EAdi and Paw were completely dissociated (that is asynchrony), such as wasted efforts, auto-triggering, multiple assist during EAdi peak (double triggering) and multiple EAdi peaks during assist, were assigned 100% error. To estimate the overall extent of asynchrony and dyssynchrony, we calculated the NeuroSync index by averaging the percentage errors for all breaths.

### Statistical analysis

The D’Agostino and Pearson test was used to test the normality of distribution. NIV-PSV_Vision_, NIV-PSV_Servo-I_ and NIV-NAVA were compared using the Friedman test with Dunn’s *post hoc* testing. Exponential regression analysis using a least squares fit was performed to test the relationship between the NeuroSync index and wasted efforts. A *P* value <0.05 was considered significant. Statistical analyses were performed with Graphpad Prism 5 (Graphpad Software, San Diego, CA, USA). Results are reported as median with interquartile ranges.

## Results

Twelve patients (one female/eleven male) were enrolled. One patient was excluded from the offline analysis due to an EAdi signal with too low an amplitude for automated patient-ventilator interaction analysis [20]. Patient characteristics and ventilator settings are shown in Tables 1 and 2, respectively. After study completion, NIV failed in two patients and invasive ventilation was required. From these two patients, one deceased.

**Table 1:** **Patient characteristics at study inclusion**

**Number**	**Age (y)**	**BMI (kg/m** ^**2**^ **)**	**FEV1 (% pred.)**	**FVC (% pred.)**	**FEV1 /FVC**	**GOLD class.**	**PF ratio**	**Reason for admission**	**Total NIV duration (days)**
1	37	25				I	316	Haemoptysis	5
2	74	23				I	308	Exacerbation COPD	3
3	68	38	45	79	42	III	185	Exacerbation COPD	3
4	67	34	69	98	70	II	176	Pneumonia	4
5	67	27	72	100	53	II	180	Exacerbation COPD	5
6	64	26	31	52	43	III	220	Trauma	3
7	58	26				II	143	Exacerbation COPD	6
8	70	28	67	90	55	II	215	Post-op lobectomy	2
9	78	22	77	88	64	II	110	Exacerbation COPD	3
10	75	17	23	61	28	IV	219	Exacerbation COPD	1
11	76	25	62	101	45	II	246	Exacerbation COPD	5

Recent lung function tests for patient 1, 2 and 7 were unavailable in our hospital, but clinical pictures of these patients were consistent with COPD and patient correspondence stated a history of COPD. BMI: body mass index; FVC: forced vital capacity; FEV1: forced expired volume in 1 second; GOLD class: Global Initiative for Chronic Obstructive Lung Disease classification; PF ratio: PaO_2_/FiO_2_.

**Table 2:** **Ventilator settings**

**Patient**	**PS level**	**PS rise time (s)**	**PS cycle off**	**NAVA level**	**NAVA**	**PEEP**	**FiO**_**2**_
	**(cmH2O)**		**(% peak flow)**	**(cmH2O/μV)**	**trigger (μV)**	**(cmH2O)**	**(%)**
1	6	0.20	30	0.1	0.5	6	70
2	10	0.20	50	0.8	0.5	8	50
3	8	0.20	70	0.4	0.5	8	50
4	5	0.20	50	0.1	1.0	7	60
5	6	0.20	50	0.1	0.5	4	40
6	5	0.05	50	5.0	0.5	5	30
7	10	0.00	50	0.2	0.5	6	55
8	6	0.20	50	0.2	0.5	6	45
9	6	0.20	50	0.2	0.5	6	70
10	8	0.00	60	0.1	0.5	5	35
11	6	0.20	50	0.2	0.5	6	40

Pressure support (PS) levels and rise time hold for both ventilators, whereas cycle-off criteria is only set for NIV-PSV_Servo-I_. The BiPAP Vision uses the Auto-Trak Sensitivity algorithm to trigger and cycle off the ventilator and cannot be set individually. PEEP and FiO2 were similar for all three ventilatory modes. FiO_2_: inspired oxygen fraction; NAVA: neurally adjusted ventilatory assist; PEEP: positive end-expiratory pressure.

### Breathing pattern and respiratory drive

Results for breathing pattern and respiratory drive are presented in Table 3. EAdi amplitude was higher with NIV-PSV_Servo-I_ compared to NIV-PSV_Vision_ (*P* <0.05). Peak airway pressure and peak flow were higher with NIV-PSV_Vision_ (*P* <0.05) compared to NIV-NAVA and NIV-PSV_Servo-I_.

**Table 3:** **Breathing pattern and respiratory drive**

	**NIV-PSV**_**Vision**_	**NIV-PSV**_**Servo-I**_	**NIV-NAVA**
Peak EAdi (μV)	25.6 (18.6 - 43.5)*	34.7 (18.8 - 49.0)	23.8 (17.1 - 48.0)
Peak airway pressure (cmH_2_O)	15.3 (13.0 - 18.5)*^#^	12.5 (10.4 - 15.2)	12.9 (11.7 - 16.0)
Inspiratory peak flow (L/min)	92.5 (72.1 - 110.0)*^#^	54.1 (46.8 - 63.2)	45.6 (38.7 - 61.1)
Neural resp. rate (breaths/min)	22.7 (17.6 - 27.0)	25.2 (18.5 - 28.2)	25.1 (18.3 - 31.7)

^*^NIV-PSV_Vision_ vs. NIV-PSV_Servo-I_ (*P* <0.05), ^#^NIV-PSV_Vision_ vs. NIV-NAVA (*P* <0.05). EAdi, electrical activity of the diaphragm; NAVA: neurally adjusted ventilatory assist; NIV: non-invasive ventilation; PSV: pressure support ventilation.

### Patient-ventilator interaction

**Figure 1:**
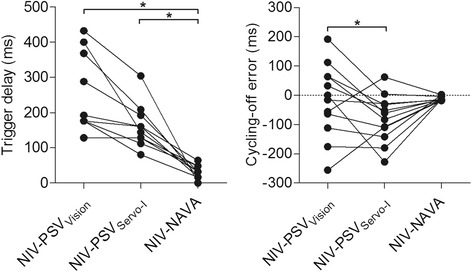
**Trigger delay (left) and cycling-off error (right) for the different ventilator modes.** Y-axis for cycle-off error: positive values indicate late cycling off, and negative values indicate early cycling off. ^*^ *P* <0.05. NAVA: neurally adjusted ventilatory assist; NIV: non-invasive ventilation; PSV: pressure support ventilation.

Figure 1 depicts median values for trigger delays and cycling-off error during each mode for all individual patients. NIV-NAVA showed lowest trigger delay compared to NIV-PSV_Vision_ and NIV-PSV_Servo-I_ (*P* <0.0001). NIV-PSV_Vision_ and NIV-PSV_Servo-I_ had comparable trigger delays, but NIV-PSV_Servo-I_ showed more early cycling off (*P <*0.05). In absolute values, NIV-PSV_Vision_ (95 ± 22 ms) and NIV-PSV_Servo-I_ (91 ± 19 ms) showed more cycling-off error compared to NIV-NAVA (12 ± 6 ms); *P* <0.05.

Patient-ventilator interaction, calculated with the NeuroSync index, was significantly higher (larger error) with NIV-PSV_Vision_ (24 (interquartile range (IQR) 15 to 30) %) and NIV-PSV_Servo-I_ (21 (IQR 15 to 26) %) compared to NIV-NAVA (5 (IQR 4 to 7) %; *P* <0.001).

**Figure 2:**
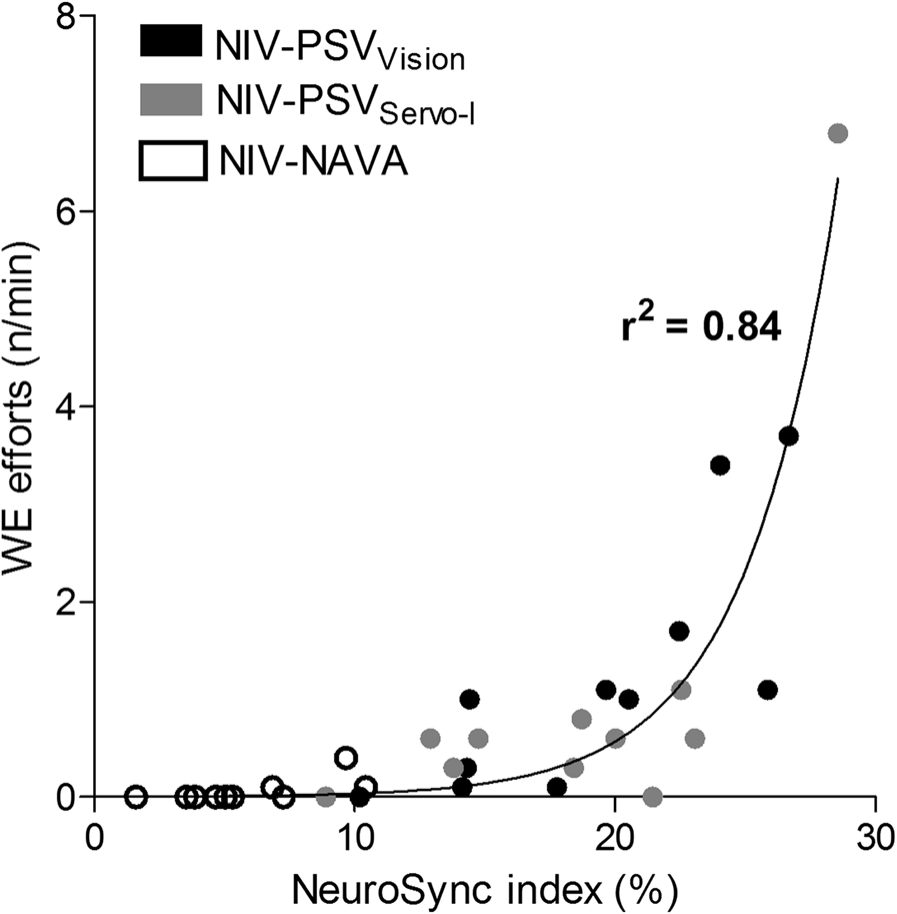
**Correlation between the number of wasted efforts and the NeuroSync index.** Note that for this regression analysis, the NeuroSync index was recalculated without wasted efforts to avoid mathematically coupled variables, and is thus consequently primarily a measure of dyssynchrony (trigger and cycle-off errors). Accordingly this correlation shows that progressive dyssynchrony, increases the number of wasted efforts. NAVA: neurally adjusted ventilatory assist; NIV: non-invasive ventilation; PSV: pressure support ventilation.

Figure 2 depicts the correlation between the number of wasted efforts and the NeuroSync index. The relationship shows as timing errors progressively increased with NIV-PSV_Servo-I_ and NIV-PSV_Vision_ a positive association with the number of wasted efforts, which was certainly more pronounced above 20% error.

**Figure 3:**
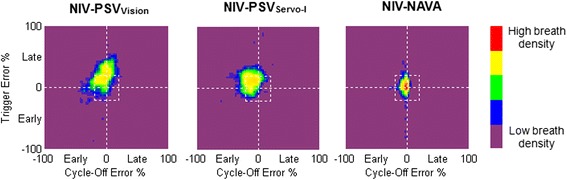
**Breath density graph for relative trigger (Y-axis) and cycling-off (X-axis) errors, for all breaths in all patients, during each ventilator mode.** The small white ‘box’ in the center of each graph indicates the limit between synchrony (neural efforts matched to assist delivery with less than 20% error - inside the box) and dyssynchrony (neural efforts poorly related to assist delivery, >20% error - outside the box). These breath-density graphs show for NIV-NAVA a concentrated breath density in the center, which should be anticipated since it is driven by EAdi. With NIV-PSV_Vision_ and NIV-PSV_Servo-I_ breaths are more spread out and have considerable proportions of dyssynchronous breaths compared to NIV-NAVA. NAVA: neurally adjusted ventilatory assist; NIV: non-invasive ventilation; PSV: pressure support ventilation.

For all three modes of NIV studied, Figure 3 shows a plot of the relative timing errors of triggering (Y-axis) versus the relative timing error for cycling off (X-axis), for every breath, in all patients. Based on the data from Figure 2, we have inserted a box suggesting ‘acceptable’ synchrony to be ≤20% of neural timings, whereas larger errors (>20%) represent dyssynchrony.

**Figure 4:**
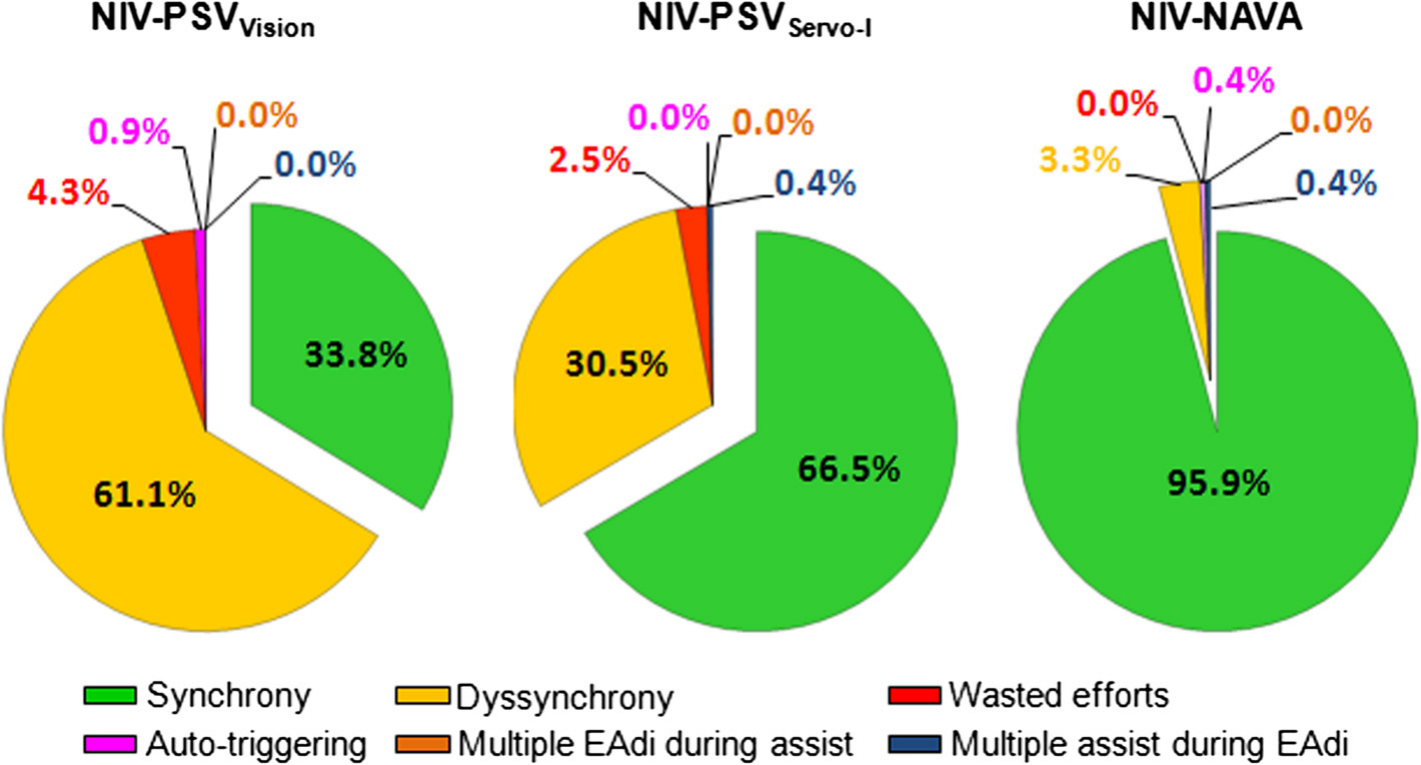
**Percentage of synchronous, dyssynchronous and asynchronous (wasted efforts, auto-triggering, multiple EAdi during assist, and multiple assist during EAdi) breaths for the different ventilator modes.** NAVA: neurally adjusted ventilatory assist; NIV: non-invasive ventilation; PSV: pressure support ventilation.

In the form of a pie chart, Figure 4 plots the distribution of synchrony (≤20% error, that is inside the box in Figure 3), dyssynchrony (>20% error, that is outside the box in Figure 3), and asynchronies for each mode. Wasted efforts were the most prevalent type of asynchrony and differed between ventilator modes (*P* <0.001). *Post hoc* analysis indicated significantly more wasted efforts with NIV-PSV_Vision_ compared to NIV-NAVA. Other asynchronies, such as multiple EAdi during assist, double triggering and auto-triggering were uncommon.

### Blood gas values and respiratory discomfort

There were no differences in blood gas values (Table 4) and respiratory discomfort with NIV-PSV_Vision_ (45 (IQR 31 to 69) mm), NIV-PSV_Servo-I_ (60 (IQR 41 to 65) mm), and NIV-NAVA (45 (IQR 33 to 75) mm).

**Table 4:** **Blood gas values**

	**NIV-PSV**_**Vision**_	**NIV-PSV**_**Servo-I**_	**NIV-NAVA**
pH	7.38 (7.36 - 7.46)	7.38 (7.36 - 7.45)	7.38 (7.36 - 7.45)
PaO_2_ (mmHg)	92 (77 - 106)	105 (84 - 113)	95 (77 - 98)
PaCO_2_ (mmHg)	44 (39 - 64)	44 (33 - 59)	41 (32 - 60)
HCO3^−^ (mmol/L)	27 (23 - 32)	26 (21 - 31)	27 (22 - 30)

NAVA: neurally adjusted ventilatory assist; NIV: non-invasive ventilation; PSV: pressure support ventilation.

## Discussion

This study provides insight into the interaction between patient and ventilator during non-invasive ventilation with different types of ventilators and modes in patients with COPD. First, we show that neurally adjusted non-invasive ventilation synchronizes assist to inspiratory effort in patients with COPD, whereas dedicated NIV ventilator or ICU ventilator pressure support modes do not ensure acceptable patient-ventilator interaction in individual patients. Second, wasted efforts increase drastically after timing errors between EAdi and airway pressure reach 20%. Third, automated analysis of patient-ventilator interaction using computer algorithms allows objective detection of patient-ventilator interaction during NIV.

### Patient-ventilator interaction

For effective unloading of the respiratory muscles with NIV, the ventilator should cycle in synchrony with the patient’s neural respiratory drive [5]. Our results are consistent with previous studies that showed improved patient-ventilator interaction with neurally compared to pneumatically controlled mechanical ventilation [15-18], however, several differences between these and the current study should be noted. First, we included only patients with COPD, which are more likely to exhibit poor patient-ventilator interaction [19]. Second, dedicated NIV-NAVA and NIV-PSV software was used instead of software for invasive ventilation in the previous studies [16,17]. This is important as the software for invasive ventilation lacks leakage compensation thereby allowing auto-triggering at high leakage. Indeed, auto-triggering up to 6 breaths/min was found with NIV-NAVA using the invasive software [16], whereas we found only up to 1 breath/min. Third, a dedicated NIV ventilator was evaluated in the present study. In bench-test comparisons, including the ventilator used in our study, PSV delivered by dedicated NIV ventilators allowed better patient-ventilator interaction than ICU ventilators with NIV algorithms [10,22]. Lastly, an automated analysis method for quantifying patient-ventilator asynchronies and the more subtle dyssynchronies was used [20], allowing a more objective detection of patient-ventilator interaction.

The present study showed a small trigger delay with NIV-NAVA, which substantially increased with NIV-PSV_Servo-I_ and NIV-PSV_Vision_. These findings agree with previous work comparing NIV-PSV_Servo-I_ and NIV-NAVA [15], but oppose a previous bench test showing longer trigger delay for NIV-PSV_Servo-I_ compared to NIV-PSV_Vision_ [22]. NAVA triggers on the increase in EAdi and thus represents the duration to increase EAdi, to process the signal and to open the inspiratory valve. Our average trigger delay of about 50 ms with NIV-NAVA is in the range previously reported for NIV-NAVA [15,16,18]. In contrast, pneumatic triggering is more complex and considerably affected by leakage, which can only be partly compensated for by dedicated NIV algorithms [7].

Synchronized termination of assist is another key component to maintain good patient-ventilator interaction. As depicted in Figure 1, NIV-PSV_Vision_ showed large inter-subject variability in early and late cycling off, whereas NIV-PSV_Servo-I_ showed primarily early cycling off. Cycling-off error in NIV-NAVA was negligible, which could be anticipated since its definition for cycling off is similar to the algorithm used to quantify cycling-off error (70% of peak EAdi). These findings agree with previous suggestions that NIV algorithms for ICU ventilators tend to increase the incidence of premature cycling off [7].

Wasted efforts are inspiratory efforts not rewarded by ventilatory assist, which can increase the work of breathing [5,6]. In the present study, 4.3% and 2.5% of inspiratory efforts were unnoticed by the ventilator for NIV-PSV_Vision_ and NIV-PSV_Servo-I_, respectively. In contrast, NIV-NAVA effectively prevented wasted efforts, confirming previous studies [15,16,18]. Furthermore, as depicted in Figure 2, we found that wasted efforts increase drastically after timing errors reach 20%. This suggests that the limits of the NeuroSync index and the definition of ‘acceptable’ synchrony should be kept below 20%, as indicated by the centered boxes in Figure 3.

### Breathing pattern and respiratory drive

EAdi in the present study was higher with NIV-PSV_Servo-I_ compared to NIV-PSV_Vision_, which is difficult to explain. Lack of difference in blood gases or respiratory rates contradict that increased EAdi with NIV-PSV was ventilation related. Premature cycling off with NIV-PSV_Servo-I_ could be a probable cause for increased EAdi, since this results in unassisted inspiration in the last part of inspiration. It should also be noted that the design of the respiratory circuit and assist delivery of the BiPAP Vision is fundamentally different from the Servo-I. For example, the BiPAP Vision system has a large intentional leakage. Consequently, from Ohm’s law it follows that higher flow is required to maintain the preset pressure level (Table 3). Higher flow might have resulted in higher CO_2_ clearance in the interface and upper airways and a consequent reduction in dead space leading to reduced respiratory drive.

### Clinical implications

Good patient-ventilator interaction is one of the key factors for clinical success of NIV, thus solving poor patient-ventilator interaction in COPD patients is of potential clinical value. In our study, we demonstrate that progressive mismatch between timing of the patient’s neural drive and the response of the ventilator is associated with increased number of wasted efforts. It is tempting to speculate that improving synchrony between patient neural effort and ventilator assist improves outcome in COPD patients, but it should be noted that our study is a short-term physiological study performed in a center with extensive experience in NIV, both with PSV and NAVA. In addition, a limitation of the present study is the limited number of patients, which hamper drawing generalized conclusions.

Differences in patient-ventilator interaction between ventilator modes did not affect blood gas values, in particular pH, and respiratory discomfort. In part, this results from the timing of study inclusion, after initial stabilization on NIV. At inclusion in the study, blood pH (around 7.38) was already increased, making it more difficult to detect changes in pH and respiratory discomfort caused by different ventilator modes. In this context it should also be mentioned that NIV modes were not performed in a random order. Nevertheless, we performed our measurement after initial stabilization on NIV making it unlikely that the patients’ respiratory status was worse at the beginning of the study than at the end. Future studies, which randomize between NAVA and PSV at admission, are necessary to ascertain whether or not improved patient-ventilator interaction in the acute phase of NIV translates to better NIV outcomes.

## Conclusions

Automated analysis of patient-ventilator interaction showed that non-invasive NAVA improves patient-ventilator interaction compared to PSV in COPD patients. Moreover, this is not different when PSV is delivered by a dedicated NIV ventilator. In addition, progressive mismatch between neural effort and pneumatic timing is strongly associated with the number of wasted efforts. Whether NAVA is more successful in correcting pH in patients with acute hypercapnic exacerbation of COPD should be addressed in future studies that randomize between NAVA and PSV at admission.

## Key messages

- Non-invasive NAVA improves patient-ventilator interaction compared to PSV in COPD patients.
- Progressive mismatch between neural effort and pneumatic timing is strongly associated with the number of wasted efforts.

## Abbreviations

COPD: chronic obstructive pulmonary disease
EAdi: electrical activity of the diaphragm
ICU: intensive care unit
IQR: interquartile range
NAVA: neurally adjusted ventilatory assist
NIV: non-invasive ventilation
Paw: airway pressure
PEEP: positive end-expiratory pressure
PSV: pressure support ventilation
